# *De Novo* Assembly and Characterization of the Transcriptome of the Chinese Medicinal Herb, *Gentiana rigescens*

**DOI:** 10.3390/ijms160511550

**Published:** 2015-05-20

**Authors:** Xiaodong Zhang, Andrew C. Allan, Caixia Li, Yuanzhong Wang, Qiuyang Yao

**Affiliations:** 1 College of Resources and Environment, Yuxi Normal University, Yuxi 653100, China; E-Mails: zxd95@126.com (X.Z.); licaixia1112@126.com (C.L.); 2 Plant and Food Research, Mt Albert Research Centre, Private Bag, Auckland 92169, New Zealand; E-Mail: andrew.allan@plantandfood.co.nz; 3 Institute of Medicinal Plants, Yunnan Academy of Agricultural Sciences, Kunming 650223, China; 4 University of Chinese Academy of Sciences, Beijing 100039, China; E-Mail: yaoqiuyang@mail.kib.ac.cn

**Keywords:** *Gentiana rigescens*, gentiopicroside, regulation, transcriptome

## Abstract

*Gentiana rigescens* is an important medicinal herb in China. The main validated medicinal component gentiopicroside is synthesized in shoots, but is mainly found in the plant’s roots. The gentiopicroside biosynthetic pathway and its regulatory control remain to be elucidated. Genome resources of gentian are limited. Next-generation sequencing (NGS) technologies can aid in supplying global gene expression profiles. In this study we present sequence and transcript abundance data for the root and leaf transcriptome of *G. rigescens*, obtained using the Illumina Hiseq2000. Over fifty million clean reads were obtained from leaf and root libraries. This yields 76,717 unigenes with an average length of 753 bp. Among these, 33,855 unigenes were identified as putative homologs of annotated sequences in public protein and nucleotide databases. Digital abundance analysis identified 3306 unigenes differentially enriched between leaf and root. Unigenes found in both tissues were categorized according to their putative functional categories. Of the differentially expressed genes, over 130 were annotated as related to terpenoid biosynthesis. This work is the first study of global transcriptome analyses in gentian. These sequences and putative functional data comprise a resource for future investigation of terpenoid biosynthesis in Gentianaceae species and annotation of the gentiopicroside biosynthetic pathway and its regulatory mechanisms.

## 1. Introduction

*Gentiana rigescens*, also named Caine gentian, belongs to Gentiana, a member of the Gentianaceae family. It is a geoherb of great importance to China’s Yunnan province. *G. rigescens* is a perennial, growing amongst hillside grasses, bushes, and trees, at relatively high altitudes. It is usually harvested in late October, with the roots being used as bulk herbs [1] and raw materials for more than 180 different Chinese traditional medicines [2]. Recent research has shown that it possesses potential functions in liver protection and immune promotion [3,4]. The main effective component of *G. rigescens* is gentiopicroside [3], which is mainly found in the vacuoles of root cells, although it is synthesized in shoots [5,6]. The content of gentiopicroside in roots was far higher than that in shoots at the flower stage [7]. In addition, there are other active components including swertiamarin, sweroside, erythricine, ursolic acid, oleanolic acid, loganic acid, gentianidine, and gentiana aldin [5,8].

In recent years, the wild resources of *G. rigescens* have declined sharply, with shortages of gentian, as demand for its use in clinical, pharmaceutical, and veterinary areas increases [1]. It has now been classified as a protected plant in China [1]. Similarly, many other *Gentiana* species have become endangered species [9]. Studies have suggested that the chromosome number of *Gentiana triflora*, *Gentiana scabra*, *Gentiana manshurica*, and other herbs containing gentiopicroside, is 2*n* = 26, while that of *Gentiana lutea* and *Gentiana punctata* is 2*n* = 40 [10]. The former three share a similar genome size (5 × 10^9^ bp/1C), approximately 33 times that of *Arabidopsis thaliana* [10,11]. Gentian genome resources are very scarce due to its large genome, genomic heterozygosity brought by distal hybridization, long growth cycle, and the lack of genetic information [10]. The Japanese gentian’s genetic linkage map was the first map of the Gentianaceae to be published, although its coverage is still low (about 1/3 genome coverage) and the phenomena of separation distortion (whereby there is unequal segregation of pairs of alleles) emerged in 30% of the molecular markers tested in progeny [10]. Therefore, the development of batches of EST-SSR (Expression Sequence Tag-Simple Sequence Repeat) molecular makers by RNA-Seq would be an improvement. In Japan, *G. scabra* and *G. triflora* are important cut and potted flowers, so research has focused on the anthocyanin biosynthesis pathway and its regulation [12,13,14]. Other studies have been on seed germination [4,15], elemental analysis [16], and active ingredient content [17,18]. However, there has been little research on the gentiopicroside biosynthesis pathway and its regulation. Recently, a seven-year breeding project of *G. rigescens*, whose goals are high yield, high gentiopicroside content, disease resistance, mechanized production, and wider planting, has been launched in Yunnan province [2].

To protect wild gentian resources as a source of plant material, a better understanding of the plant’s biology and growth is required. Transcriptome research provides a method of fast, high-throughput, comprehensive interpretation of the plant’s genome information, including new gene function information, the biosynthesis of the active ingredients and their regulation, and germplasm evaluation and expansion [19].

The objective of this research was to compare the transcriptomes of the leaf and root of *G. rigescens*, using Illumina Hiseq2000. To determine genes involved in the gentiopicroside biosynthesis pathway and its regulatory mechanism, transcripts from leaves and roots of gentian were isolated, quantified, sequenced, and annotated. The results described here will aid further functional genomic studies in gentian.

## 2. Results and Discussion

### 2.1. Sequencing and Assembly

To determine the transcriptomes of the leaves and roots of *G. rigescens*, two sequencing libraries were prepared and sequenced with the Illumina paired-end technique. As a result, over 50 million clean reads per library were obtained after cleaning and quality checks were performed. The sequencing data quality assessments are shown in Table 1. The error rate of both root and leaf is 0.03% (Q20 and Q30 are over 96% and 90%, separately), indicating a high quality of sequence. The sequencing raw data has been deposited into the Short Reads Archive (SRA) database under the accession number SRP027253.

**Table 1:** The quality assessment of the sequencing data.

Item	Sample	Raw Reads	Clean Reads	Clean Bases (G)	Error (%)	Q20 (%)	Q30 (%)	GC (%)
Leaf	Leaf_1	57,802,913	56,289,486	5.63	0.03	97.97	93.09	43.00
	Leaf_2	57,802,913	56,289,486	5.63	0.03	97.48	92.61	43.04
Root	Root_1	53,933,882	50,596,096	5.06	0.03	97.02	90.79	43.23
	Root_2	53,933,882	50,596,096	5.06	0.03	96.59	90.34	43.31

Leaf_1, Root_1, Leaf_2, Root_2: The left and the right reads, separately; Raw reads: Statistical raw sequence data with four lines as a unit, to sum the sequence number of each file; The Clean Reads of the Leaf or Root were the sum of the left and right reads. Error rate: Bases error rate; Q20 and Q30: The percentages of the bases whose Phred values were more than 20 and 30, separately.

The clean reads were combined and assembled by using the Trinity program, which has been shown to be an excellent assembler for *de novo* transcriptome assembly from short-read RNA-Seq data [20]. Assembled sequences were subjected to cluster using the Trinity algorithm. As a result, 191,541 contigs clustered into 78,433 Trinity components (mean size = 743 bp, N50 = 1365 bp). Each Trinity component defines a collection of transcripts that are most likely to be derived from the same locus (except a portion from very closely related paralogs) [20,21]. This component was defined as a unigene and the longest transcript in each component was used to represent the corresponding unigene in this study. After removal of 1716 (2.2% of total) contaminant unigene sequences from non-plant species (see Materials and Methods), a transcriptome of 76,717 unigenes with a total size ~57.7 Mb was established for *G. rigescens*. The sequences of the unigenes (longer than 200 bp) were deposited in the NCBI (National Center for Biotechnology Information) Transcriptome Shotgun Assembly Sequence Database (TSA) according to its standard criteria (downloaded from the BioProject: PRJNA211794, Accession Number: GDAB00000000). The full list of transcript sequences is also available upon request.

### 2.2. Assembly Assessment

**Figure 1:**
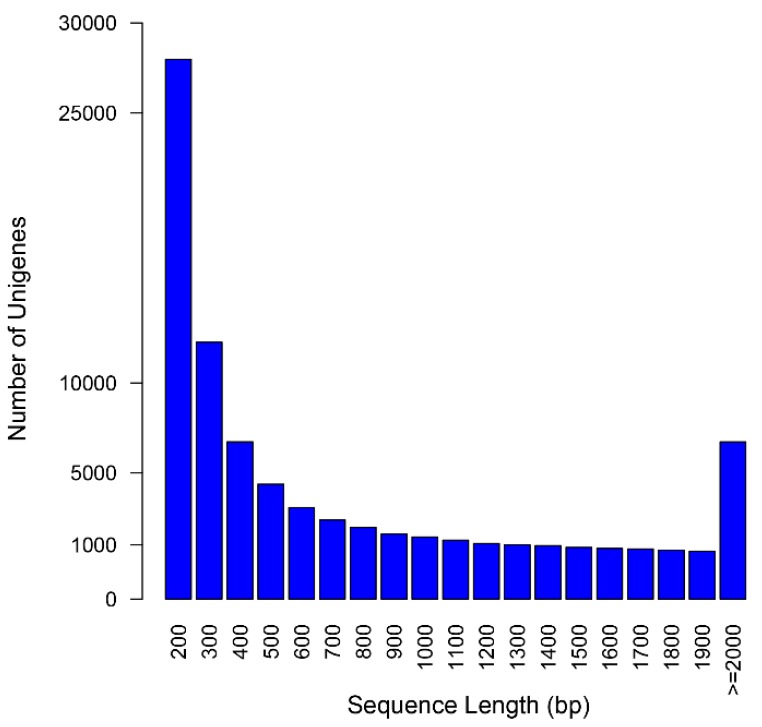
Length distribution frequency of the unigenes in *G. rigescens*.

By comparing our results to *Gentiana* sequences downloaded from the NCBI (Available online: http://www.ncbi.nlm.nih.gov), we demonstrated that the assembly succeeded in constructing a large amount of transcripts with desirable length. Of 43,611 *Gentiana* sequences, 33,773 (77.4%) sequences were represented in our assembly (Megablast, *E*-value was 10^−9^), among which 23,908 (70.8%) sequences were matched with more than 80% identity and 80% coverage. RNA-Seq reads were mapped back to the assembly to calculate the proportion of reads assembled, indicating a statistic report comparable to other *de novo* assemblies. The total alignment rate was 92.72% (Table 2), and 78.3% of the mapped paired-reads aligned concordantly, which showed good physical evidence of sequence contiguity. Transcript length (such as N50, average length) is another broadly used parameter to overview the quality of the transcriptome assembly. As shown in Figure 1, the unigenes ranged from 201 to 16,728 bp, with a mean length of 753 bp and an N50 length of 1384 bp, which is comparable to similar RNAseq reports. Thus, we have successfully constructed a desirable assembly from Illumina paired-end sequencing.

**Table 2:** Summary of the transcriptome assembly of *G. rigescens*.

Item	Contigs	Unigenes
Total number	189,576	76,717
Total length (bp)	228,624,912	57,734,637
Mean length (bp)	1206	753
N50 (bp)	1996	1384
GC content (%)	39.7	39.5
Number of length ≥ 500 bp	120,525	29,795
Number of length ≥ 1000 bp	81,919	16,332
Reads mapping rate (%)	92.72	-

### 2.3. Gene Function Annotation and Classification

**Figure 2:**
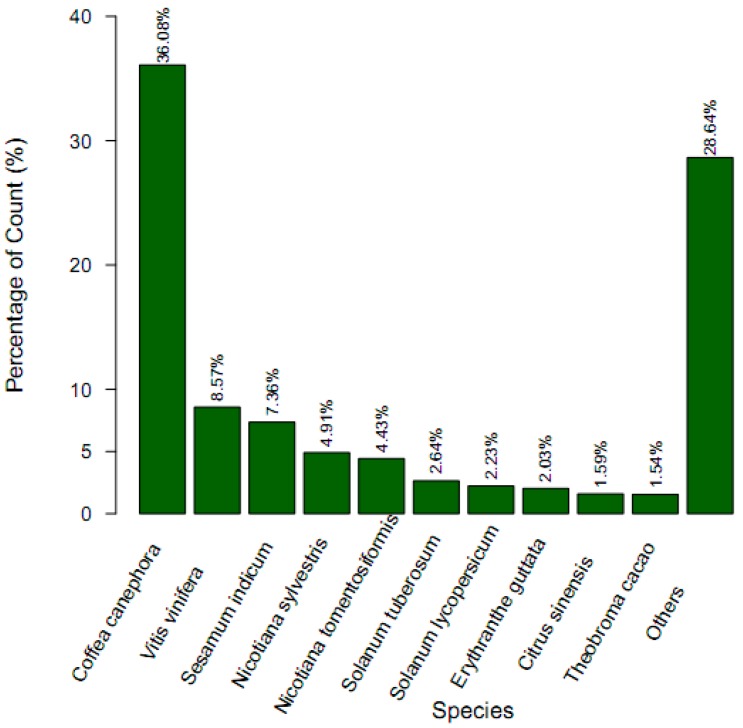
Species distribution of the top BLAST (Basic Local Alignment Search Tool) hits for each unigene against NR (Non-redundant) database.

All the 76,717 assembled putative unigenes were aligned using the BLAST program against the NR, NT, Swiss-Prot and COG databases with the E-value cutoff of 10^−5^. A total of 33,855 unigenes were annotated, accounting for 44.13% (Table 3). Among them, 26,686 unigenes (34.78%) showed high homology, with sequences in the NR database, 24,371 unigenes (31.77%) matched to protein sequences in TAIR10, and 18,627 unigenes (24.28%) showed homology with known genes in SwissProt. The detailed results are shown in Table 3 and Table S1–S3. Based on the top-hit species distribution of the homology result against NR databases, 26,361 unigenes (92.08%) showed high homology with sequences from land plants, among which the highest matches were to genes from *Coffea canephora* (36.08%), followed by *Vitis vinifera* (8.57%), and *Sesamum indicum* (7.36%) (Figure 2).

**Table 3:** Statistics result of gene annotation.

Item	Number of Unigenes (*n*)	Percentage (%)
Annotated in NR	26,686	34.78
Annotated in NT	8158	10.64
Annotated in TAIR10	24,371	31.77
Annotated in KEGG	7998	10.43
Annotated in SwissProt	18,627	24.28
Annotated in PFAM	23,287	30.35
Annotated in GO	26,494	34.53
Annotated in KOG/COG	10,524	13.72
Annotated in all Databases	3019	3.94
Annotated in at least one Database	33,855	44.13
Total queries/unigenes	76,717	100

**Figure 3:**
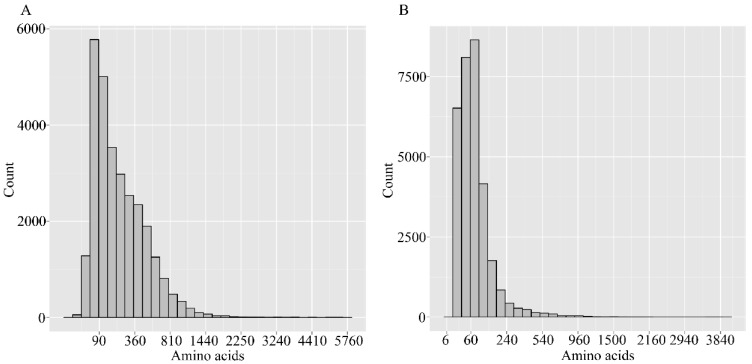
Length distributions of predicated peptides. (**A**) Predicated by BLAST; (**B**) Predicated by ESTScan program (Available online: http://www.ch.embnet.org/software/ESTScan.html). The abscissa represents the peptide length, while the ordinate represents thce number of the corresponding number.

Putative protein sequences were obtained by translating using a standard codon table. The CDSs of unigenes that did not match the above databases were predicted with the ESTSCAN software. The gene length distribution is shown in Figure 3. The length of peptides predicted by BLASTp ranges from 60–810, while that of ESTSCAN are 30–240.

**Figure 4:**
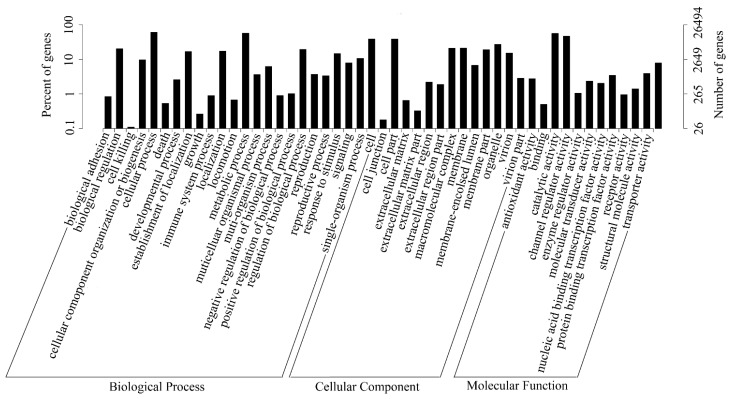
GO classification map. The abscissa represents the next level GO term of the three GO categories, while the ordinate represents the number of genes annotated into the corresponding term, and its proportion of the total number of annotated genes.

In this study, all unigenes were searched against the GO database. Out of 76,717 unigenes, 26,494 were successfully annotated and classified into three GO categories: biological process, cellular component, and molecular function, and assigned to 56 functional groups (Figure 4). As shown in Figure 4, assignments which fell under cellular component ranked the highest, followed by biological process, and molecular function. In the biological process category, “cellular process” (16,075, 60.67%) and “metabolic process” (15,223, 57.46%) were the two most representative subcategories. In the cellular component category, unigenes related to “cell” (10,308, 38.91%) and “cell part” (10,282, 38.81%) were dominant, while in the molecular function category, the majority of unigenes were involved in “binding” (14,903, 56.25%) and “catalytic activity” (12,326, 46.52%). These results suggested that many kinds of enzyme pathways were active in gentian.

**Figure 5:**
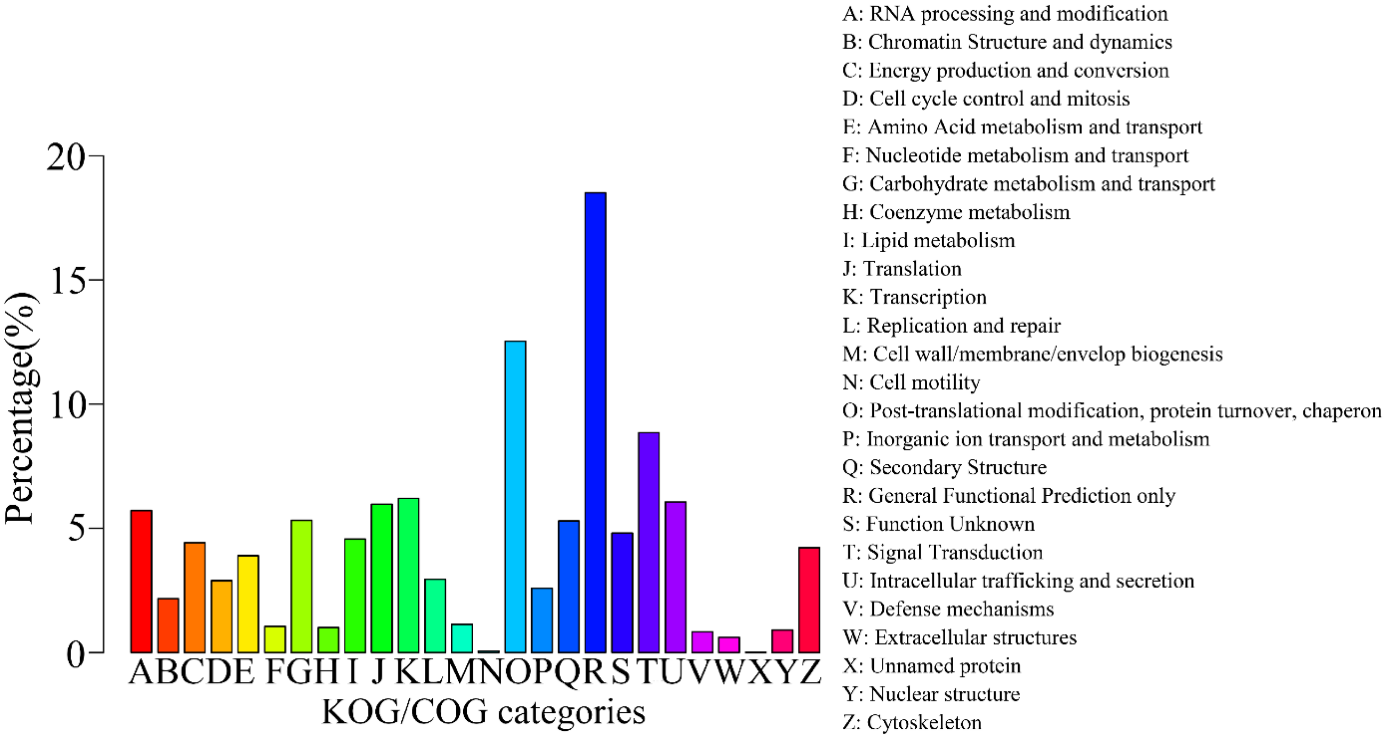
KOG/COG classification map. The abscissa represents 26 group names of KOG/COG, while the vertical axis represents the number of genes annotated into the group and its proportion of total number of annotated genes.

A total of 10,524 sequences were classified into 26 KOG/COG (Clusters of Orthologous Groups of proteins) groups (Figure 5), where “General function prediction only” category accounted for the most frequent group (1948, 18.51%), with the second largest group being “Post-translational modification, protein turnover, chaperon” (1319, 12.53%), followed by “Signal transduction” (932, 8.86%) and “Translation” (654, 6.21%). These results showed that in the flower stage of gentian, the protein translation and signal transduction are active.

**Figure 6:**
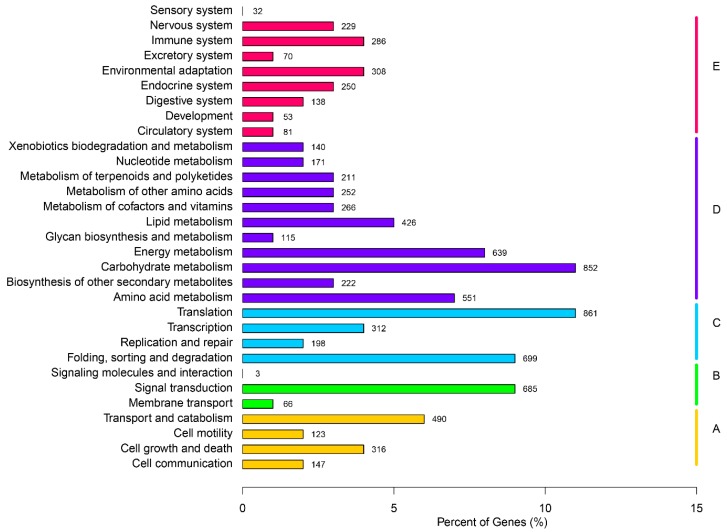
KEGG classification map. The ordinate is the name of the pathway, while the abscissa is the proportion of genes belonging to this pathway. These genes were divided into five branches: (A, Cellular Processes; B, Environmental Information Processing; C, Genetic Information Processing; D, Metabolism; E, Organismal Systems.) according to the metabolic pathway they participated in.

The KEGG (Kyoto Encyclopedia of Genes and Genomes) metabolic system is a group of metabolic maps which represents current understanding of biomolecular interaction networks. In order to determine the active pathways in flowering gentian, KEGG assignments of all unigenes were performed. Referencing the 7998 unigenes of *G. rigescens* through the KEGG database predicted a total of five categories (level 1, cellular processes, environmental information processing, genetic information processing, and metabolism and organismal systems), 31 sub-categories (level 2, Figure 6) and 238 pathways (level 3). Unigenes identified as related to the “Translation” (861, 10.77%), “carbohydrate metabolism” (852, 10.65%), “Folding, sorting and degradation” (699, 8.74%) and “Signal transduction” (685, 8.56%) were the top four representative pathways (Figure 6). Unigenes counts for “Terpenoid backbone biosynthesis”, “Monoterpenoid biosynthesis”, “Diterpenoid biosynthesis”, “Sesquiterpenoid and triterpenoid biosynthesis”, and “Ubiquinone and other terpenoid-quinone biosynthesis” were 55, 5, 22, 21, and 31, separately. These results indicated that the terpenoid pathways were active in flowering gentian, and the corresponding genes would be candidate genes for gentiopicroside biosynthesis.

**Figure 7:**
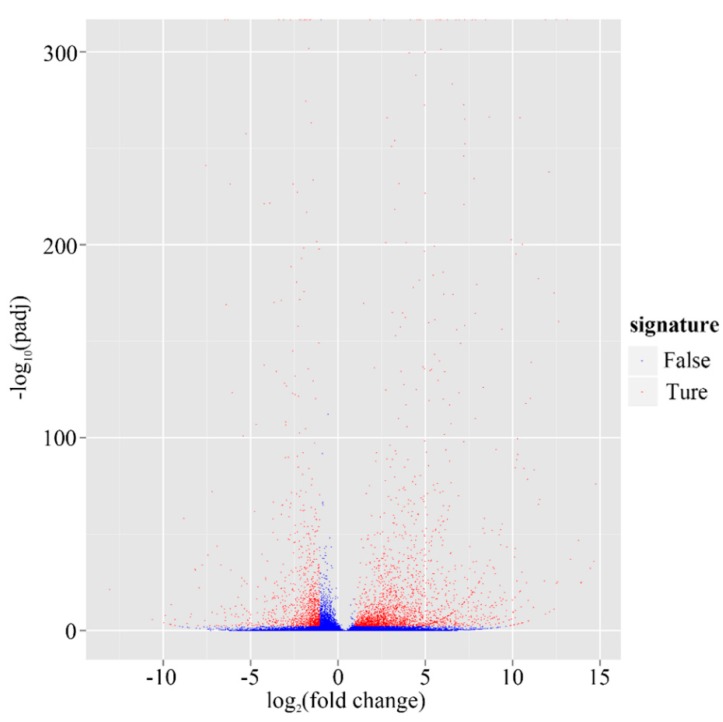
Volcano plot of Leaf *ves.* Root in *G. rigescens*. The abscissa represents changes of gene expression (Leaf *vs.* Root). The ordinate represents the statistical significance of change of the amount of gene expression. The less *p*-value, the more −log_10_(*p* value), and the more significance. The scattering dots represent genes, while the blue dots show genes without significant differences and *vice versa* for red dots.

Gene expression was calculated using the RPKM method, which takes into account both sequencing depth and gene length effects on read count [22]. On the basis of the applied criteria *q*-value <0.005 and log_2_(foldchange) >1, 3306 genes (4.31% of all genes) were identified as significantly differentially expressed genes (DEGs) between these two tissues, which comprised 2204 up-regulated genes (accounting for 67%) and 1102 down-regulated genes (33%) in leaves (Figure 7, Table S4). The log_2_(fold changes) ranged from one to 15. Not surprisingly, among these DEGs, most were related to photosynthesis, for example, ribulose-1,5-bisphosphate carboxylase/oxygenase (RuBisCO)-a, a key enzyme of the Calvin-Benson cycle of autotrophic CO_2_ assimilation [23], chloroplast chlorophyll a/b-binding protein, photosystem II 22 kDa protein gene, and chloroplastic ferredoxin genes, were all up-regulated over 10-fold in leaves compared to roots. The terpenoid biosynthesis related genes, such as geranyl diphosphate synthase (GPPS), geraniol synthase (GES), geraniol 10-hydroxylase (G10H), and iridoid oxidase (IO), four key enzymes involving monoterpene biosynthesis, were all up-regulated over 10-fold in leaves compared to roots.

Of the down-regulated genes, a late embryogenesis abundant (LEA) protein, was 13-fold higher in roots than in leaves. Late Embryogenesis Abundant (LEA) proteins are a group of hydrophilic proteins with a high content of glycine, and are associated with stress tolerance in plants and animals through protecting enzymatic function and inhibition of aggregation in dehydration, heat, and salt stress [24,25]. In *Arabidopsis thaliana*, overexpression of *LEA14* enhances salt stress tolerance [26]. Ectopic expression of *ZmLEA5C* in tobacco and yeast enhances their tolerance to osmotic and low temperature stresses [27]. A calcium-dependent protein kinase (CDPK) gene involved in plant defense responses [28] was nine-fold higher in roots than in leaves. Previous research suggests that CCaMK is an important component of the symbiosis signaling pathway [29,30,31,32,33,34]. In *Zea mays*, calcium/calmodulin-dependent protein kinase (ZmCCaMK) is required for abscisic acid (ABA)-induced antioxidant defense systems [35]. A high affinity nitrate transporter [36] was eight-fold higher in roots than in leaves. In higher plants, there are two nitrate uptake systems, the high and low affinity transporter systems, and the high affinity nitrate transporter functions when the nitrate concentration is low [37,38].

### 2.4. Putative Genes Involved in the Terpenoid Backbone Biosynthesis and Gentiopicroside Biosynthetic Pathways

**Figure 8:**
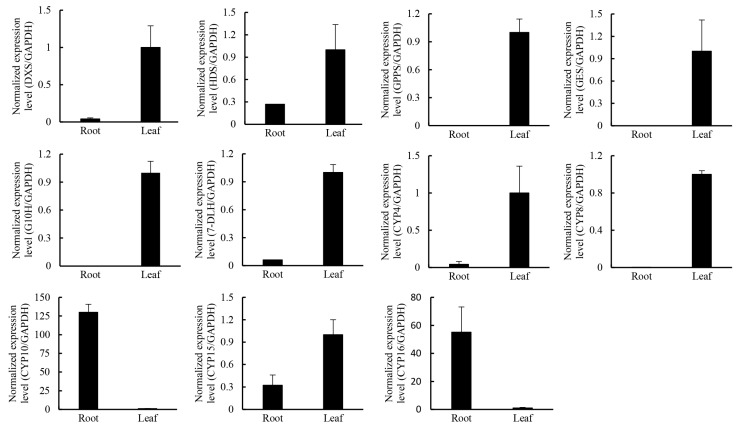
The expression pattern of three selected MEP pathway genes and *CYP* genes in roots and leaves in *G. rigescens*. Means ± SE; each qRT-PCR was biologically repeated three times.

Terepenoids, including monoterpenoids, diterpenoids, chlorophyls, carotenoids, abscisic acid, cytokinin gibberellins, sterols, sesquiterpenoids, and ubiquinones, are all closely related with the terpenoid backbone biosynthesis [39,40]. The terpenoid backbone is derived from the universal precursor, isopentenyl diphosphate (IPP), and its allylic isomer, dimethylallyldiphosphate (DMAPP), which are derived from the mevalonate (MVA) and/or the methylerythritol phosphate (MEP) pathways [41] (Figure S1). Transcripts encoding the enzymes involved in the MVA and MEP pathways were searched against the unigenes and transcripts present in our database (Table 4). In general, transcripts of MVA and MEP pathway genes were more abundant in leaves, as revealed by much higher numbers of reads of 3-hydroxy-3-methylglutaryl-CoA reductase (*GrHMGR*), 5-diphosphomevalonate decarboxylase (*GrMVD*), Isopentenyl diphosphate isomerase (*GrIDI*), 1-deoxy-Dxylulose 5-phosphate synthase (*GrDXS*), 1-deoxy-d-xylulose-5-phosphate reductoisomerase (*GrDXR*), 2-*C*-methyl-d-erythritol 2,4-cyclodiphosphate synthase (*GrMCS*), and 4-hydroxy-3-methylbut-2-enyl diphosphate synthase (*GrHDS*) genes in leaves than in roots (Table 5, (Figure S2). qRT-PCR (quantitative Reverse Transcription-Polymerase Chain Reaction) results showed that the selected genes *GrDXS1*, *GrHDS*, and *GrIDI1* were more abundant in leaves (Figure 8). These results support the observation that gentiopicroside is synthesized in shoots and allocated to the roots [6].

**Table 4:** Expression of putative genes in MVA and MEP biosynthesis pathways.

Pathway	Gene Name	Unigene	RPKM in Leaf	RPKM in Root
MVA	*AACT1*	comp81670_c0	50.65	52.76
	*AACT2*	comp86403_c0	13.16	38.43
	*HMGS*	comp1196622_c0	0.00	0.49
	*HMGR1*	comp87249_c0	27.14	3.53
	*HMGR2*	comp92954_c0	35.56	17.08
	*HMGR3*	comp4296_c0	0.25	0.57
	*HMGR4*	comp25979_c0	0.57	0.04
	*HMGR5*	comp114241_c0	5.72	0.00
	*MK*	comp83300_c0	20.74	16.19
	*PMK1*	comp371052_c0	0.55	0.48
	*PMK2*	comp82309_c1	2.21	1.23
	*PMK3*	comp82309_c0	3.94	2.21
	*PMK4*	comp92698_c0	8.08	11.20
	*MVD1*	comp86107_c0	51.01	39.76
	*MVD2*	comp73189_c0	0.55	0.58
	*IDI1*	comp81822_c0	114.79	89.95
	*IDI2*	comp67360_c0	37.65	31.50
	*IDI3*	comp92050_c0	49.18	2.79
MEP	*DXS1* *	comp87916_c0	45.34	1.45
	*DXS2*	comp89290_c0	10.85	3.64
	*DXS3*	comp93517_c0	48.69	35.56
	*DXR*	comp92087_c3	123.96	107.04
	*MCT*	comp67067_c0	36.76	8.28
	*MCS*	comp91375_c0	56.61	36.13
	HDS *	comp94424_c0	151.28	77.99
	*HDR1*	comp87777_c0	114.07	97.97
	*HDR2*	comp509208_c0	0.00	0.91
	*HDR3*	comp1116482_c0	0.00	0.36

* These genes were selected for qRT-PCR.

**Table 5:** Expression of putative genes in secoiridoid biosynthesis pathways.

Gene Name	Unigene	RPKMs in Leaf	RPKMs in Root
*GPPS1* *	comp57663_c0	47.61	0.04
*GPPS2*	comp79818_c0	53.25	8.76
*GES* *	comp45416_c0	66.71	0.06
*G10H*	comp95013_c1	304.45	1075.00
*G10H* *	comp59018_c0	128.37	0.15
*G10H*	comp67411_c0	6.01	10.68
*G10H*	comp84881_c0	41.33	89.38
*G10H*	comp74631_c0	31.33	70.85
*G10H*	comp64598_c0	15.04	42.75
*G10H*	comp42518_c0	3.07	13.76
*G10H*	comp89824_c0	14.26	28.73
*G10H*	comp67522_c0	11.48	15.46
*G10H*	comp92644_c0	164.26	389.80
*G10H*	comp67745_c0	13.73	25.95
*G10H*	comp77398_c0	4.41	7.13
*G10H*	comp51247_c0	0.00	2.77
*G10H*	comp67165_c0	16.78	27.36
*G10H*	comp67799_c0	0.00	0.90
*G10H*	comp63189_c0	0.10	1.55
*G10H*	comp76700_c0	1.03	2.28
*G10H*	comp42518_c0	3.07	13.76
*8HGO*	comp93669_c0	161.97	2.80
*8HGO*	comp53753_c1	81.29	1.37
*8HGO*	comp53753_c2	121.86	0.92
*8HGO*	comp76718_c0	65.89	77.05
*8HGO*	comp90961_c0	5.52	2.34
*8HGO*	comp92998_c0	219.14	245.73
*SLS*	comp94595_c0	504.62	20.96
*SLS*	comp94064_c5	368.11	50.11
*SLS*	comp84511_c0	27.90	35.43
*SLS*	comp81016_c0	0.59	0.46
*SLS*	comp85876_c0	318.25	345.13
*SLS*	comp67629_c0	2.53	3.00
*SLS*	comp54852_c0	0.08	1.74
*SLS*	comp55055_c0	0.56	2.47
*SLS*	comp61732_c0	0.75	2.06
*SLS*	comp93282_c0	143.24	185.59
*SLS*	comp281520_c0	0.49	0.75
*SLS*	comp87446_c0	22.83	39.78
*SLS*	comp41718_c0	0.09	4.01
*SLS*	comp167742_c0	0.43	1.74
*SLS*	comp94107_c0	121.99	222.06
*SLS*	comp49781_c0	0.00	0.70
*SLS*	comp90874_c0	14.95	24.36
*SLS*	comp212851_c0	1.23	0.83
*SLS*	comp73409_c0	0.00	0.72
*SLS*	comp87446_c0	22.83	39.78
*SLS*	comp73685_c0	0.95	2.11
*SLS*	comp76988_c0	4.19	4.57
*SLS*	comp103080_c0	4.73	6.98
*SLS*	comp81659_c0	3.97	6.59
*IS*	comp85292_c0	64.52	0.00
*IO*	comp84741_c0	361.42	0.00
*7-DLGT*	comp82018_c0	65.59	0.00
*7-DLH* *	comp94064_c5	368.11	50.11
*CYP1*	comp84741_c0	361.42	0.00
*CYP2*	comp89478_c0	33.35	2.23
*CYP3*	comp108293_c0	10.24	0.09
*CYP4* *	comp92783_c1	27.39	8.86
*CYP5*	comp80146_c0	17.02	3.45
*CYP6*	comp83496_c0	46.67	160.59
*CYP7*	comp94595_c0	504.62	20.96
*CYP8* *	comp97650_c0	29.44	2.77
*CYP9*	comp90225_c0	16.82	1.16
*CYP10* *	comp80525_c0	0.06	15.35
*CYP11*	comp92026_c0	60.41	4.19
*CYP12*	comp68870_c0	3.04	31.70
*CYP13*	comp85931_c0	59.63	6.58
CYP14	comp79921_c0	75.72	33.66
*CYP15* *	comp80492_c0	19.18	6.02
*CYP16* *	comp95479_c0	99.29	14.68
*CYP17*	comp90874_c0	14.95	24.36

* These genes were selected for qRT-PCR.

Monoterpenes are mainly synthesized in the plastid using geranyl diphosphate (GPP) as a precursor [41]. Following the formation of the acyclic terpenoid structural building blocks, terpene synthases act to generate the main terpene carbon skeleton, and the cytochrome P450 (CYP450) superfamily may catalyze these reactions [42]. However, CYP450enzymes form one of the largest gene families, with over 127 plant cytochrome P450-families being described [43]. The number of CYP450s involved in gentiopicroside biosynthesis remains unclear. Most terpenoid-related CYP450s are members of the CYP71clade, a large group that comprises CYP450s involved in the metabolism of specialized compounds [44]. In the *G. rigescens* transcriptome data, 169 putative CYP450s transcripts were identified that belong to 60 families as dictated by the standard CYP family categories (Table S5), and the majority are CYP716B2 family members (20 unigenes).

**Figure 9:**
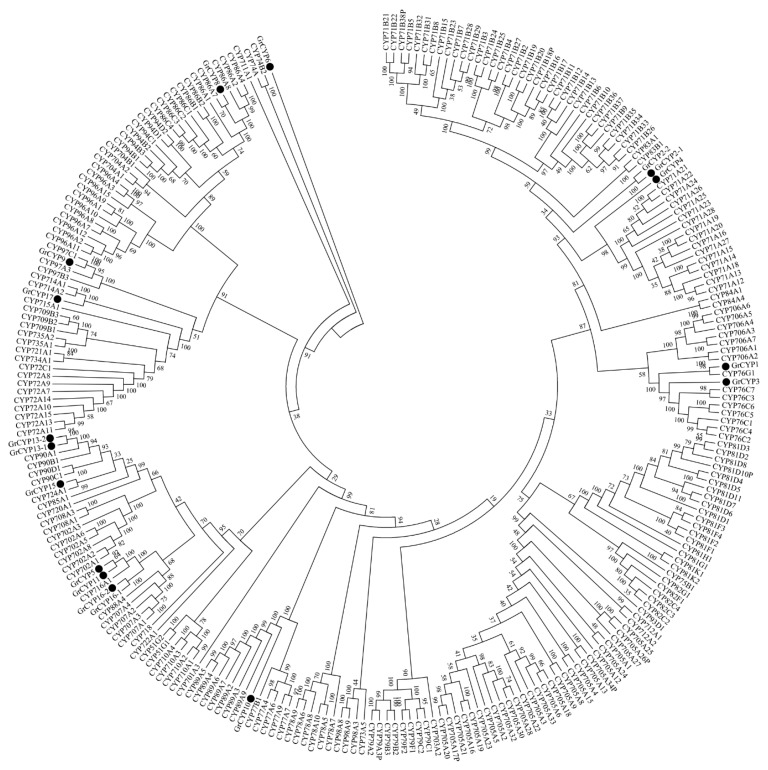
Phylogenetic analysis of CYP450s from *G. rigescens.* Amino acid sequences were aligned using the CLUSTALX2 program, and evolutionary distances were calculated using MEGA6 software with the Neighbor-Joining statistical method and Poisson model. The bootstrap replications were set to 1000. The GenBank accession numbers of the sequences are GrCYP1 (KP218047), GrCYP2-1 (KP218048), GrCYP2-2 (KP218049), GrCYP3 (KP218050), GrCYP4 (KP218051), GrCYP5 (KP325125), GrCYP6 (KP218052), GrCYP8 (KP325126), GrCYP9 (KP218053), GrCYP450-10 (KJ829649), GrCYP11 (KP218054), GrCYP13-1 (KP218055), GrCYP13-2 (KP218056), GrCYP15 (KJ829650), GrCYP16-1 (KP218057), GrCYP16-2 (KP218058), and GrCYP17 (KF941188). The sequences of *Arabidopsis thaliana* come from TAIR (Available online: https://www.arabidopsis.org).

In the differential expression analysis, several *CYP450* genes were screened out. Some which had Open Reading Frames (ORFs) with a BLASTX score of *E*-value <10^−5^, were then verified by RT-PCR and sequencing. Phylogenetic analysis of the deduced protein sequences with P450s from *Arabidopsis thaliana* revealed that five of them (GrCYP4, GrCYP5, GrCYP11, GrCYP16, and GrCYP17) belong to the CYP71 clan (Figure 9). qRT-PCR results showed that the selected genes *GrCYP4*, *GrCYP8*, and *GrCYP15* were highly expressed in leaves, however, *GrCYP10* and *GrCYP16* genes were more abundant in roots (Figure 8).

In the secoiridoid biosynthesis pathway, IPPs and DMAPPs are condensed into GPP by GPPS, which is then converted to geraniol by GES. Geraniol is catalyzed to 8-oxogeraniol by geraniol 8-oxidase (G8O, also named G10H) [42,45], and then to 8-oxogeranial by 8-hydroxygeraniol oxidoreductase (8HGO, also named 10HGO) [42,45]; 8-oxogeranial is sequentially catalyzed into loganin via several steps including iridoid synthase (IS), IO, 7-deoxyloganetic acid glucosyltransferase (7-DLGT), 7-deoxyloganic acid hydroxylase (DL7H), loganic acid *O*-methyltransferase (LAMT), and secologanin synthase (SLS) [45,46,47]. In *Catharanthus roseus*, G10H, SLS, and DL7H were three important enzymes of the monoterpenoid biosynthesis pathway [48,49,50]. In the *G. rigescens* transcriptome, there were annotated two *GrGPPS*s, one *GrGES*, 18 *GrG10H*s, six *Gr8HGO*s, 24 *GrSLS*s, one *Gr7DLH*, one *GrIO*, one *GrIS*, and one *Gr7-DLGT*, but no sequence annotated as *GrLAMT*. Of interest was that *GrIO*, *GrIS*, *Gr7-DLGT*, and *GrCYP1* were only expressed in leaves. Differential expression analysis identified five genes *GrGPPS1*, *GrGES*, *GrG10H*, *Gr7DLH*, and *GrCYP1*, which were upregulated 10 times more in leaves than in roots (Table 5). Meanwhile, there were three *Gr8HGO*s, one *GrSLS*, one *GrIS*, one *Gr7-DLGT*, one *GrCYP3*, and one GrCYP7, whose expression was five times higher in leaves than in roots (Table 5). However, the expression of one *GrSLS* and one *GrCYP10* was downregulated more than five times in leaves compared to roots (Table 5). qRT-PCR results showed that *GrGPPS1*, *GrGES*, *GrG10H*, and *Gr7DLH* genes were more highly expressed in leaves than in roots (Figure 8), which suggested that secologanin was mainly synthesized in leaves. These results provide further evidence for gentiopicroside synthesis in shoots [6].

### 2.5. Candidate Transcription Factors Involved in Regulating the Terpenoid Biosynthetic Pathway

TFs play key roles in controlling gene expression [51], and the controlled transcription of biosynthetic genes is one major mechanism regulating secondary metabolite production in plant cells [52,53,54]. The floral terpenoids of snapdragon appear to be derived exclusively from the MEP pathway in plastids, and this pathway controls precursor levels for GPPS, which in turn is transcriptionally regulated [55]. In our *G. rigescens* unigene dataset, 7176 unigenes were annotated as transcription factors (Table S6), including bHLH (349), AP2-EREBP (172), WRKY (141), MYB (129), bZIP (115), and GRAS (94) family members. Among these, most were expressed in both root and leaf tissues, with 80 showing a significantly higher expression level in leaves than in roots (Table 6, Table S7).

**Table 6:** Summary of transcription factor unigenes of *G. rigescens*.

TF Family	Number of Genes Detected	Up-Regulated in Leaves (log_2_(Fold_Change) > 2)	Up-Regulated in Roots (log_2_(Fold_Change) > 2)
HLH	349	26	5
AP2-EREBP	172	20	4
WRKY	141	17	1
MYB	129	7	2
bZIP	115	3	4
GRAS	94	7	1
Total	1000	80	17

**Figure 10:**
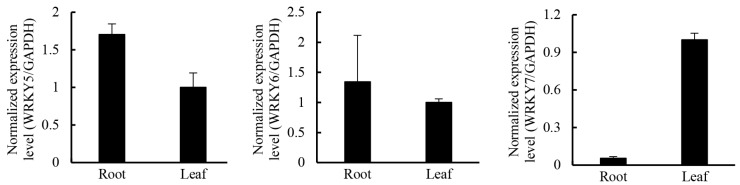
The expression pattern of three selected *WRKY* genes in roots and leaves in *G. rigescens*. Means ± SE; each qRT-PCR was biologically repeated three times.

Members of the WRKY transcription factor family have been shown to regulate secondary metabolism pathways [56]. In *Gossypium arboreum*, GaWRKY1 regulates sesquiterpene biosynthesis via activation of δ-cadinene synthase (CAD1-A) [57]. In *Coptis japonica*, the biosynthesis of berberine is controlled by CjWRKY1 [58]*.* In tomato trichomes, terpene synthase are controlled by SlMYC1 and SlWRKY73 [59]. In *Catharanthus roseus*, CrWRKY1, a regulator in biosynthesis of terpenoid indole alkaloids, interacts with transcription factors, including ORCA3, CrMYC, and ZCTs, to play a role in determining the root-specific accumulation of serpentine [60,61]. In *Nicotiana attenuata*, biosynthesis of diterpene glycosides are regulated by WRKY3 and WRKY6 [62]. In leaves of *Artemisia annua*, AaWRKY1 activated the expression of the majority of artemisinin biosynthetic genes, including *AaADS* and *AaHMGR* [63]. In the present analysis, 141 unigenes were annotated as WRKY family transcription factors, of which 17 were more highly expressed in leaves than in roots (Table 6). qRT-PCR results showed that *GrWRKY7* genes were more highly expressed in leaves than in roots, while it was the opposite for *GrWRKY5* and *GrWRKY6* (Figure 10). Thus, *GrWRKY7* is a good candidate to study in the regulation of the biosynthesis of gentiopicroside.

## 3. Experimental Section

### 3.1. Plant Materials and RNA Isolation

**Figure 11:**
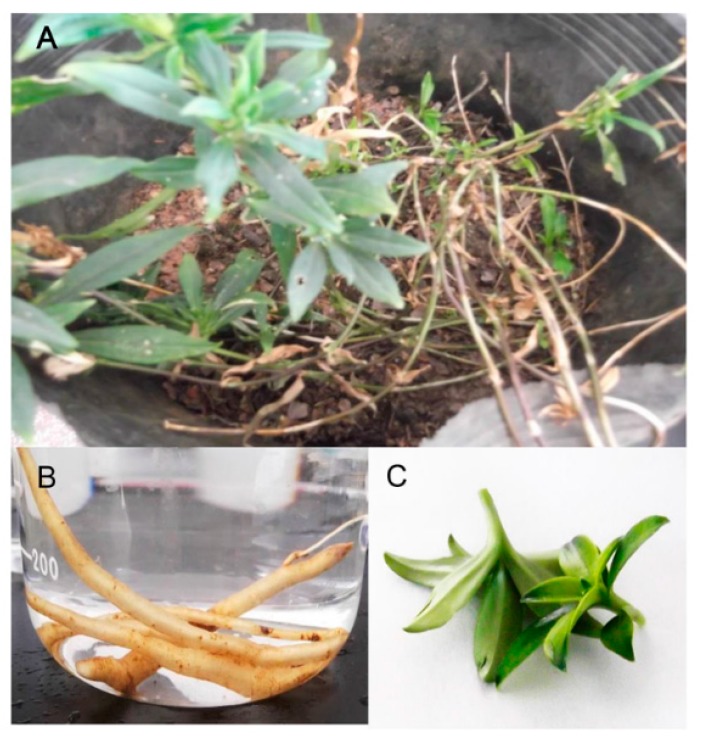
Plant materials of *G. rigescens*. (**A**) *G. rigescens* used in this article; (**B**,**C**) are roots and leaves used in experiments for sequencing.

The cultivated variety of *G. rigescens* was grown in pots with humus soil and yellow soil mixed in a 1:1 ratio. The fresh roots and leaves were collected from 3-year-old flowering gentian plants in October 2012 (Figure 11). To reduce biological bias, material of three individual plants was pooled to give 1 g of roots and 1 g of leaf samples. All samples were immediately frozen in liquid nitrogen and stored at −80 °C.

Total RNA of each sample (three plants mixed) was isolated by Illumina TruSeq™ RNA Sample Preparation Kit (RS-122-2001). RNA degradation and contamination were monitored on 1% agarose gels. RNA purity was checked using the NanoPhotometer^®^ spectrophotometer (IMPLEN, Westlake Village, CA, USA). RNA concentration was measured using Qubit^®^ RNA Assay Kit in Qubit^®^ 2.0 Flurometer (Life Technologies, Carlsbad, CA, USA). RNA integrity was assessed using the RNA Nano 6000 Assay Kit of the Bioanalyzer 2100 system (Agilent Technologies, Palo Alto, CA, USA).

### 3.2. Transcriptome Sample Preparation for Sequencingc

The construction of a cDNA library and the following sequencing procedures were as in Zhu *et al*. [64].

### 3.3. Data Filtering

Raw data (raw reads) of fastq format were firstly processed through in-house perl scripts (available upon request). In this step, clean reads were obtained by standard quality control criteria to remove all of the reads which meet any one of the following parameters: (1) The reads that aligned to adaptors with no more than two mismatches; (2) The reads with more than 10% unknown bases (N bases); (3) The reads with more than 50% of low-quality bases (quality value ≤ 5) in one read. At the same time, Q20, Q30, GC-content, and sequence duplication level of the clean data was calculated. All the downstream analyses were based on clean data with a high quality of sequencing.

### 3.4. Transcriptome Assembly and Contamination Sequences Filtering

As there are few reference sequences available for Gentianaceae, the reads for unigenes of both root and leaf were assembled together. The left files (read1 files) from all libraries/samples were pooled into one left.fq file, and right files (read2 files) into one right.fq file, both in FastQ format. Transcriptome assembly was accomplished based on the pooled paired-end reads files (left.fq and right.fq) using Trinity software (Version 2012-10-05) [20] with min_k-mer_cov set to 2 and all other parameters settings as default. The following processes are referred to Shu *et al.* [65].

Contaminant sequence level was investigated according to species distribution based on protein similarity searching against NR protein databases. Coding sequences from Non-land-plant species were identified and discarded using a previously described taxonomy-based method [66]. Contaminant sequence from major plant pathogens, and human and other microorganisms (including bacteria, virus, and fungi) was investigated using the stand-alone version of DeconSeq [67].

### 3.5. Gene Functional Annotation

To assign putative gene function, unigenes were searched against the NR (NCBI non-redundant protein sequences), NT (NCBI nucleotide sequences), TAIR10, PFAM (Protein family; Available online: http://pfam.sanger.ac.uk/), and Swiss-Prot (A manually annotated and reviewed protein sequence database; Available online: http://www.ebi.ac.uk/uniprot/) databases using BLAST software with an *E*-value cutoff of 10^−5^ [68]. Hmmerscan was adopted for PFAM annotation, and Blast2GO was used for GO annotation (Gene Ontology; Available online: http://www.geneontology.org/) [69] with the same *E*-value. To evaluate the completeness of the library and the efficacy of the annotation process, the annotated sequences were searched for the possible functions involved in KOG/COG (Available online: http://www.ncbi.nlm.nih.gov/COG/) classifications. To determine which pathways are active in leaves and roots, the annotated sequences were mapped to the reference pathways in KOG/COG, KO (KEGG Ortholog database; Available online: http://www.genome.jp/kegg/).

### 3.6. Differential Expression Analysis

The calculation of unigene expression used the RPKM method (Reads per kb per Million reads) [70]. Gene expression levels were estimated by RSEM [71] for each sample: (1) Clean data were mapped back onto the assembled transcriptome; (2) Readcount for each gene was obtained from the mapping results.

Differential expression analysis and GO enrichment analysis of leaves *vs.* roots was referred to Lv *et al*. [72]. To figure out the transcription factor families existing in leaves and roots, the transcript sequences were aligned against the Plant Transcription Factor Database with BLASTX and a cutoff of *E*-value < 10^−6^ [73].

### 3.7. KEGG Enrichment Analysis

KEGG pathway enrichment analysis of the DEGs was done using KOBAS [74].

### 3.8. Real-Time PCR Analysis

DNase I-treated total RNA of root and leaf was converted into first-strand cDNA by the use of PrimeScript RTase (Takara, Tokyo, Japan). qRT-PCRs were performed in an ABI7000 Fluorescence Quantitative PCR Instrument (Applied Biosystems, Foster City, CA, USA) using a SuperReal PreMix Plus Kit (Tiangen, China). The PCR condition was: 95 °C for 3 min; 95 °C for 15 s; 60 °C for 30 s. Each reaction was repeated three times. Glyceraldehyde-3-phosphate dehydrogenase (GAPDH) was chosen as the internal reference gene. The 2^−ΔΔ*C*t^ method was adopted for the relative gene expression. The primers used are listed in Table S8.

## 4. Conclusions

Next generation sequencing of RNA has now replaced microarrays as the preferred method for gene expression profiling. One key advantage of this method is that it enables examination of the transcriptome of non-model organisms [75]. Despite the Chinese traditional herb *G. rigescens* being used for thousands of years, the biosynthesis pathway and regulation of its main effective component, gentiopicroside, remains unknown. Few genetic or genomic studies have been performed. The results presented here addresses this by using the Illumina Hiseq2000 platform to identify sequences and transcript abundance levels of genes expressed in developing roots and leaves of *G. rigescens*. These sequences provide a starting point for further investigation of gentiopicroside biosynthesis, and include the 3306 unigenes from diverse pathways that were differentially expressed between root and leaf. The results represent a genetic resource for *G. rigescens*, and may serve as the foundation for further genomic research on *G. rigescens* and its relatives.

## Supplementary Materials

Supplementary materials can be found at http://www.mdpi.com/1422-0067/16/05/11550/s1.
