# Supplementary material for: De Novo Assembly and Characterization of the Transcriptome of the Chinese Medicinal Herb, Gentiana rigescens
Source: Int J Mol Sci. 2015 May 20;16(5):11550–73. doi: 10.3390/ijms160511550 (PMC4463717; doi:10.3390/ijms160511550)
Supplement: Supplementary file 1 [file ijms-16-11550-s001.zip › ijms-82646-Supplementary Information/ijms-82646-Supplementary Information-Figures.pdf]

Supplementary Information

Abbreviations:

AACT, acetyl-CoA acetyltransferase;
HMGS, hydroxymethylglutaryl-CoA synthase;
HMGR, 3-hydroxy-3-methylglutaryl-coenzyme A reductase;
MK, mevalonate kinase;
PMK, phosphomevalonate kinase;
MVD, diphosphomevalonate decarboxylase;
DXS, 1-deoxy-D-xylulose-5-phosphate synthase;
DXR, 1-deoxy-D-xylulose-5-phosphate reductoisomerase;
MCT, 2-*C*-methyl-D-erythritol 4-phosphate cytidylyltransferase;
MCS, 2-C-methyl-D-erythritol 2,4-cyclodiphosphate synthase;
CMK, 4-diphosphocytidyl-2-*C*-methyl-D-erythritol kinase;
HDS, 4-hydroxy-3-methylbut-2-enyl diphosphate synthase;
IDS, 4-hydroxy-3-methylbut-2-enyldiphosphate reductase (isopentenyl/dimethylallyl diphosphate synthase);
IDI, isopentenyl-diphosphate delta-isomerase;
GPPS, geranyl diphosphate synthase;
CYP450, cytochrome P450;
G10H, geraniol 10-hydroxylase;
10HGO, 10-hydroxygeraniol oxidoreductase;
G8O, geraniol 8-oxidase;
8-HGO, 8-hydroxygeraniol oxidoreductase;
IS, iridoid synthase;
IO, iridoid oxidase;
7-DLGT, 7-deoxyloganetic acid glucosyltransferase;
7-DLH, 7-deoxyloganic acid hydroxylase;
LAMT, loganic acid *O*-methyltransferase;
SLS, secologanin synthase (TIF).

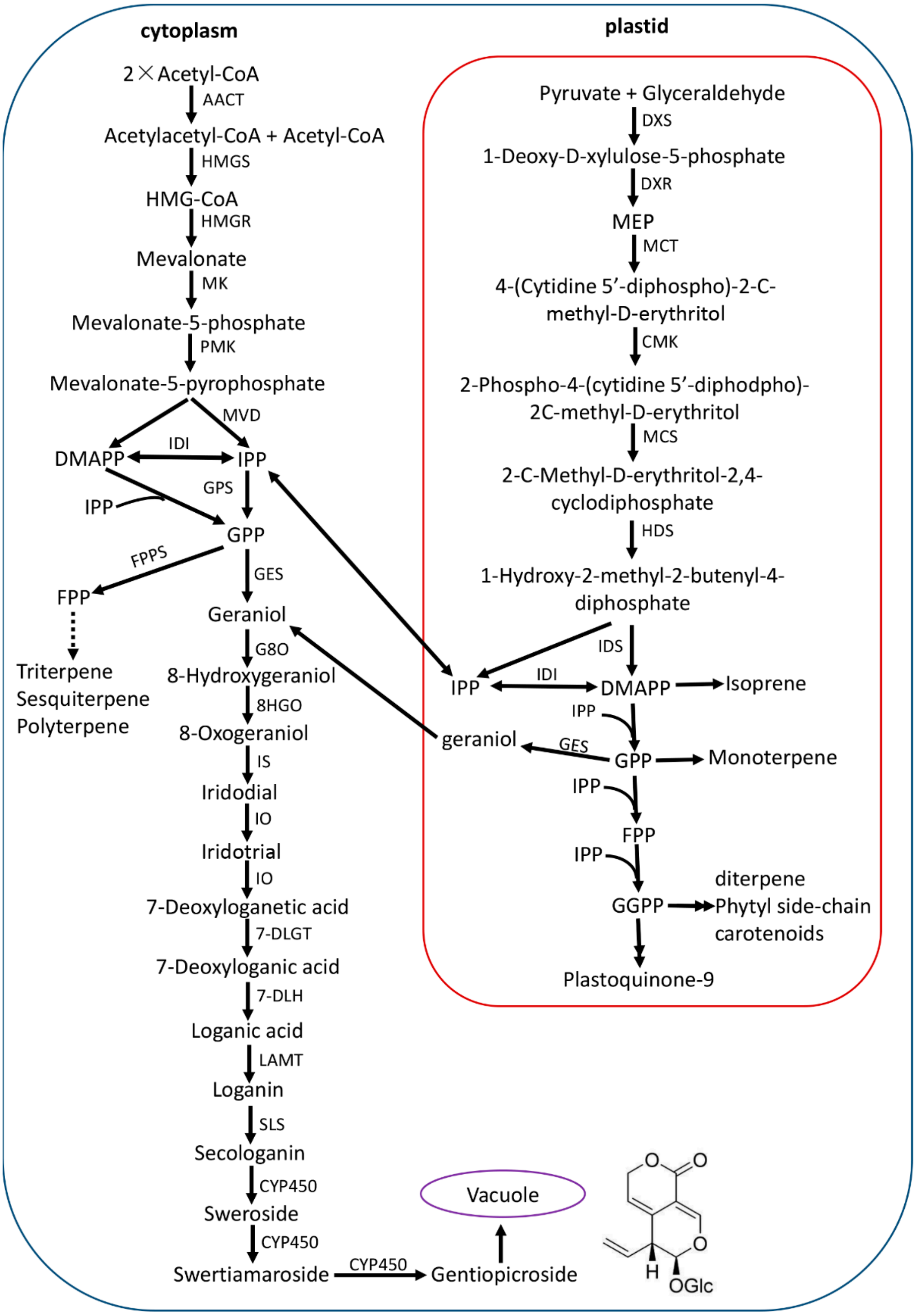

Figure S1. Putative biosynthesis pathway of gentiopicroside.

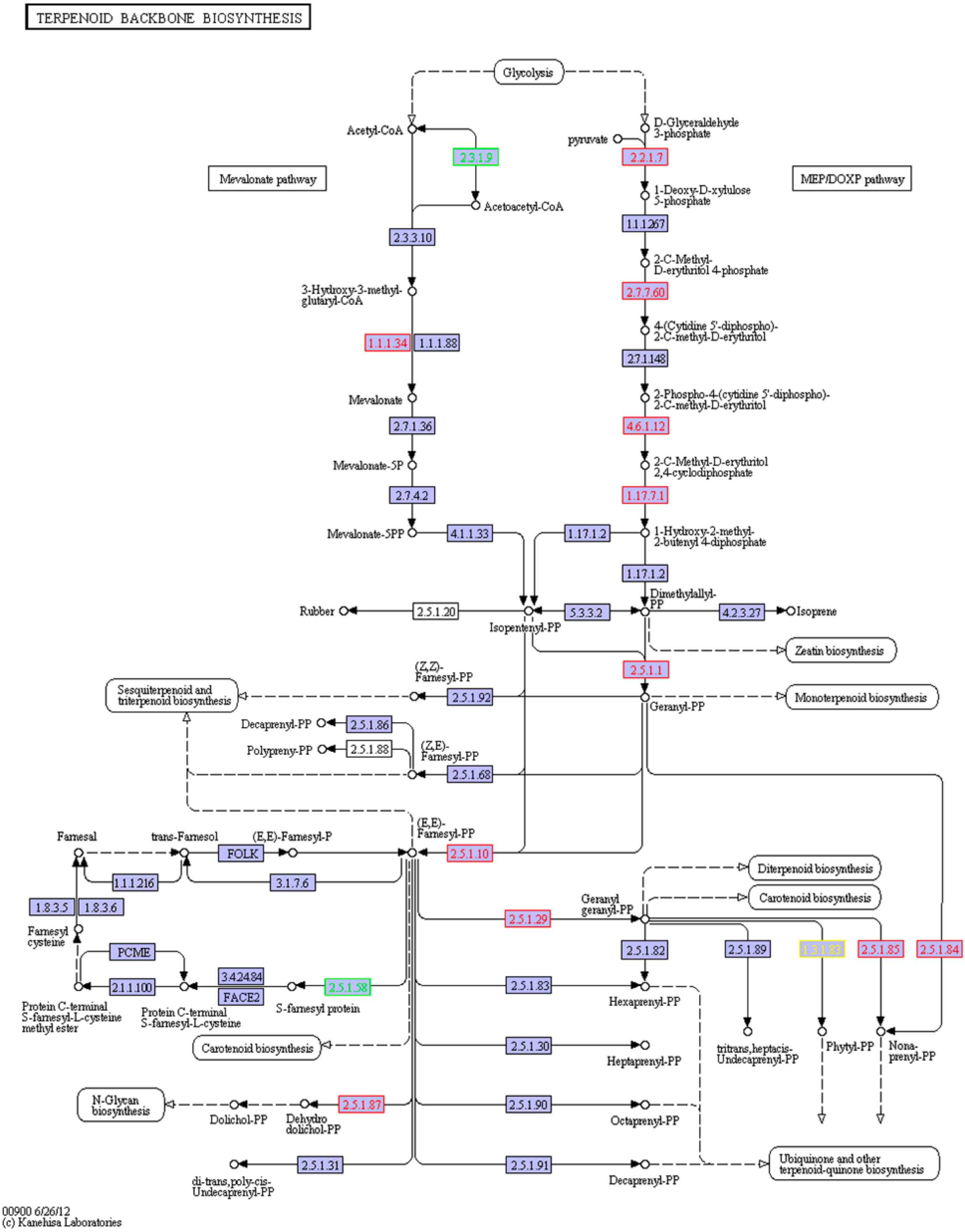

Figure S2. KEGG enriched pathway of terpenoid backbone biosynthesis (TIF).
